# Cervical cancer prognosis and related risk factors for patients with cervical cancer: a long-term retrospective cohort study

**DOI:** 10.1038/s41598-022-17733-8

**Published:** 2022-08-17

**Authors:** Jina Li, Gaoming Liu, Jiayou Luo, Shipeng Yan, Ping Ye, Jie Wang, Miyang Luo

**Affiliations:** 1 Xiangya School of Public Health, Central South University, No. 238 Shang Ma Yuan Ling Road, Changsha, 410008 Hunan China; 2 Hunan Cancer Hospital and The Affiliated Cancer Hospital of Xiangya School of Medicine, Central South University, Changsha, 410013 Hunan China

**Keywords:** Cancer epidemiology, Cancer prevention

## Abstract

This study aims to explore the recurrence rate and overall survival for patients with cervical cancer after the first treatment and the related risk factors. A retrospective cohort study was conducted on cervical cancer patients enrolled in a cancer specialist hospital in Hunan Province, China from January 1992 to December 2005 and followed up until December 2010. Kaplan–Meier survival analysis was used to estimate the cumulative recurrence rate, and Cox proportional hazards model was utilized to identify risk factors associated with prognosis. A total of 4358 patients were enrolled with a median follow-up of 7.4 years (range 5–19 years), and 372 (8.5%) patients had cancer recurrence. The cumulative recurrence rate showed a rapid increase from 3.8% in the first year after discharge to 8.0% in the fifth year, and the recurrence rate remained relatively stable afterward reaching 9.7% and 10.8% in the 10th and the 15th year, respectively. The median time to recurrence was 15.5 months with an IQR of 5.5–40.0 months. The Cox regression showed that miscarriage, clinical stage, and treatment received were significantly associated with cervical cancer recurrence after adjustment for confounders. Patients with recurrence showed a significantly higher risk for mortality than those without recurrence (HR 2.79, 95% CI 2.42–3.22). This study depicted the long-term recurrence rate and survival after recurrence for patients with cervical cancer after the first treatment, and reported time to recurrence and risk factors related to recurrence. These findings may provide important evidence for designing targeted interventions for the treatment of cervical cancer.

## Introduction

Cervical cancer is the most common malignant tumor in the female reproductive system, and it is one of the leading causes of death in women globally^1^. According to the 2020 Global Cancer Statistics Report, there were about 604,127 new cases of cervical cancer and 341,831 deaths caused by cervical cancer worldwide in 2020, accounting for 6.5% and 7.7% of the total number of cancer incidences and deaths in women, respectively^2^. Although there are standardized treatment methods for cervical cancer, patients after treatment often face the dilemma of recurrence^3^. Once the recurrence happens, the patient is left with very limited treatment options and a poor prognosis^4,5^. Thus, the prevention of cervical cancer recurrence has become a huge challenge in clinical practice.

Reducing the recurrence of cervical cancer requires an improved understanding of the actual recurrence rate, time to recurrence, and its related risk factors. Previous studies reported that the recurrence rate of cervical cancer varied in different populations (approximately 6.4–21.1%)^6–10^. To our knowledge, only a few studies focused on the pattern of recurrence rate and survival after recurrence in China^11–13^. However, most of these studies had a small sample size and a short period of observation, or were based on data from clinical trials which may limit their generalizability. In addition, most of the studies lacked in-depth research on the time to recurrence^14,15^, such as the analysis of the time to recurrence for patients with different age groups, parity, clinical stages, and treatment received. Moreover, despite that previous studies reported several risk factors of cervical cancer recurrence and prognosis, including patient's age, ethnicity, pathological type, clinical stage, lymph node metastasis, tumor size, and treatment received^16,17^, there is still controversy about certain risk factors, for instance, some studies reported inconsistent results for age, pathological type, and differentiation^6,18,19^.

This study aims to explore the long-term recurrence rate, time to recurrence, and survival after recurrence for patients with cervical cancer after the first treatment and identify the potential risk factors, using a large retrospective cohort, which may provide a scientific basis for the effective prevention and treatment of cervical cancer recurrence.

## Results

### Characteristics of the study population

From January 1992 to December 2005, a total of 4374 subjects diagnosed with cervical cancer and completed the initial treatment in the study hospital were identified. After excluding 16 subjects who were lost to follow-up, we included 4358 subjects with a mean age of 46.5 years in the final analyses. The mean follow-up duration was 7.4 years with a range from 5 to 19 years. In this study, 46.6% of subjects were in clinical stage I, 40.8% of subjects were in clinical stage II, and 12.6% of subjects were in clinical stage III and IV (Table 1). In addition, 3864 (92.2%) subjects had squamous cell carcinoma, 261 (6.2%) subjects had adenocarcinoma, and 68 (1.6%) subjects had other types of cervical cancer. We found that 479 (12.0%) subjects were well-differentiated, 3332 (83.7%) subjects were moderately differentiated, 172 (4.3%) subjects were poorly differentiated/ undifferentiated.

**Table 1:** Patient characteristics.

	Overall	Recurrence: No	Recurrence: Yes	*p* value
N	4358	3986	372	
Age (years, mean (SD))	46.46 (10.25)	46.57 (10.30)	45.34 (9.67)	0.028
**Age group (years, %)**				0.116
< 40	1218 (27.9)	1107 (27.8)	111 (29.8)	
40–59	2636 (60.5)	2406 (60.4)	230 (61.8)	
≥ 60	504 (11.6)	473 (11.9)	31 ( 8.3)	
**Parity (times, %)**^**a**^				0.015
0–2	2040 (46.8)	1843 (46.2)	197 (53.0)	
≥ 3	2318 (53.2)	2143 (53.8)	175 (47.0)	
**Miscarriage (times, %)**				0.003
0	1824 (41.9)	1694 (42.5)	130 (34.9)	
1–2	1806 (41.4)	1646 (41.3)	160 (43.0)	
≥ 3	728 (16.7)	646 (16.2)	82 (22.0)	
**Clinical stage (%)**^**b**^				0.051
I	1936 (46.6)	1798 (47.2)	138 (39.8)	
II	1694 (40.8)	1536 (40.3)	158 (45.5)	
III	511 (12.3)	462 (12.1)	49 (14.1)	
IV	13 ( 0.3)	11 ( 0.3)	2 ( 0.6)	
**Pathological type (%)**^**c**^				0.383
Squamous cell carcinoma	3864 (92.2)	3530 (92.2)	334 (91.8)	
Adenocarcinoma	261 ( 6.2)	240 ( 6.3)	21 ( 5.8)	
Others	68 ( 1.6)	59 ( 1.5)	9 ( 2.5)	
**Level of differentiation (%)**				0.507
Well	479 (12.0)	440 (12.1)	39 (11.2)	
Moderate	3332 (83.7)	3041 (83.7)	291 (83.4)	
Poor/undifferentiated	172 (4.3)	153 (4.2)	19 (5.4)	
**Treatment received (%)**^**d**^				< 0.001
Surgery only	262 (6.0)	253 (6.4)	9 (2.4)	
Surgery plus adjuvant therapy	1974 (45.4)	1805 (45.4)	169 (45.4)	
Radiotherapy only	1545 (35.5)	1432 (36.0)	113 (30.4)	
Chemotherapy plus radiotherapy	526 (12.1)	456 (11.5)	70 (18.8)	
Chemotherapy only	44 (1.0)	33 (0.8)	11 (3.0)	
**Surgery methods (%)**^**e**^				0.143
No	2122 (51.0)	1928 (50.6)	194 (55.1)	
Radical trachelectomy	93 (2.2)	83 (2.2)	10 (2.8)	
Simple hysterectomy	72 (1.7)	70 (1.8)	2 (0.6)	
Radical hysterectomy	1752 (42.1)	1613 (42.3)	139 (39.5)	
Others	125 (3.0)	118 (3.1)	7 (2.0)	
**Lymph node metastasis (%)**^**f**^				0.008
No	1804 (85.2)	1664 (85.9)	140 (78.2)	
Yes	313 (14.8)	274 (14.1)	39 (21.8)	
**Radiotherapy (%)**				0.568
No	432 ( 9.9)	392 ( 9.8)	40 (10.8)	
Adjuvant	1855 (42.6)	1706 (42.8)	149 (40.1)	
Initial	2071 (47.5)	1888 (47.4)	183 (49.2)	
**Chemotherapy (%)**				< 0.001
No	3122 (71.6)	2912 (73.1)	210 (56.5)	
Adjuvant	666 (15.3)	585 (14.7)	81 (21.8)	
Concurrent	526 (12.1)	456 (11.4)	70 (18.8)	
Initial	44 (1.0)	33 (0.8)	11 (3.0)	
**Year of admission (%)**				< 0.001
1992–1995	573 (13.1)	548 (13.7)	25 ( 6.7)	
1996–2000	1104 (25.3)	1027 (25.8)	77 (20.7)	
2001–2005	2681 (61.5)	2411 (60.5)	270 (72.6)	

^a^28 missings; ^b^204 missings; ^c^165 missings. ^d^7 missings; ^e^194 missings; ^f^based on biopsy after surgery.

Around half of the subjects received surgical treatment, with 6% receiving surgery only and 45.4% receiving surgery plus adjuvant therapy; 35.5% of subjects received radiotherapy only; 12.1% of them received chemotherapy plus radiotherapy; only 1% of subjects received chemotherapy only. When stratified by clinical stage, we observed that 8.1% and 84.2% of subjects in clinical stage I received surgery only and surgery plus adjuvant therapy, respectively. 17.3% of subjects in clinical stage II received surgery plus adjuvant therapy, 62.9% of them received radiotherapy only, and 18.3% of them received chemotherapy plus radiotherapy. Among subjects in clinical stage III/IV, 65.1% of subjects received radiotherapy only, and 28.8% of them received chemotherapy plus radiotherapy (Supplemental Table 1).

A total of 372 subjects had a cancer relapse during the follow-up period. Subjects with recurrence had significantly younger age, lower parity, and a greater number of miscarriages, and they were more likely to have lymph node metastasis and received chemotherapy (*P* < 0.05).

### Cumulative rate and time to recurrence

**Figure 1:**
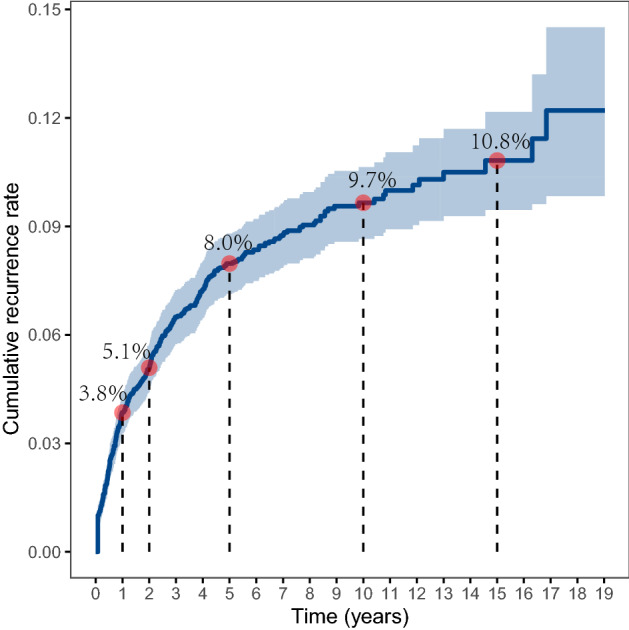
The overall cumulative recurrence rate.

**Figure 2:**
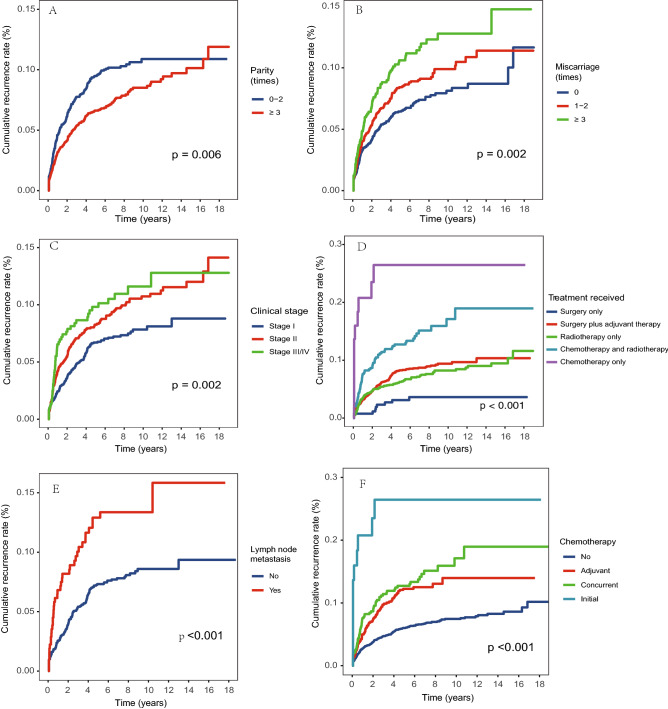
Cumulative recurrence rate by (**A**) parity; (**B**) miscarriage; (**C**) clinical stage; (**D**) treatment received; (**E**) lymph node metastasis; (**F**) chemotherapy.

The overall cumulative recurrence rate was 3.8% in the first year after discharge and increased to 5.1% in the second year, and 8.0% in the fifth year. The increasing trend slowed down afterward and remained relatively stable at around 10% after ten years (Fig. 1). The median time to recurrence for the 372 subjects during follow-up was 15.5 months (IQR 5.5, 40.0) (Table 2). There were significant differences in the recurrence rate when stratified by parity, miscarriage, clinical stage, treatment received, lymph node metastasis, and chemotherapy received (*P* < 0.05, Fig. 2), and the average time to recurrence was significantly shorter for subjects with lower parity, a greater number of miscarriages, higher clinical stage, lymph node metastasis, and received chemotherapy, especially for those with initial chemotherapy.

**Table 2:** Time to recurrence in the study population (N = 372).

Variables	Recurrence time (months)			*p* value^a^
	Median	IQR		
Overall	15.5	5.5	40.0	
**Age group (years)**				0.120
< 40	22.1	5.0	43.6	
40–59	14.2	6.0	39.7	
≥ 60	17.3	8.8	34.7	
**Parity (times)**				0.007
0–2	14.0	5.3	33.4	
≥ 3	20.0	5.9	46.4	
**Miscarriage (times)**				0.003
0	17.2	5.9	42.1	
1–2	15.8	5.4	39.6	
≥ 3	14.9	5.6	36.2	
**Clinical stage**				0.002
I	23.9	7.9	45.7	
II	15.8	5.8	41.6	
III/IV	9.1	5.8	23.6	
**Pathological type**				0.300
Squamous cell carcinoma	15.1	5.4	42.1	
Adenocarcinoma	24.1	11.4	34.1	
Others	23.8	11.3	27.8	
**Level of differentiation**				0.340
Well	19.8	5.2	55.2	
Moderate	16.7	5.6	40.5	
Poor/undifferentiated	9.5	1.2	25.7	
**Treatment received**				< 0.001
Surgery only	28.6	24.5	40.0	
Surgery plus adjuvant therapy	22.1	6.4	44.6	
Radiotherapy only	13.2	5.9	50.4	
Chemotherapy plus radiotherapy	11.0	5.6	30.5	
Chemotherapy only	1.0	1.0	6.1	
**Surgery methods**				0.060
No	11.4	5.3	34.3	
Radical trachelectomy	6.4	1.3	24.3	
Simple hysterectomy	18.7	13.7	23.6	
Radical hysterectomy	23.2	7.5	45	
Others	17	6.3	30.1	
**Lymph node metastasis**				0.001
No	23.9	8.9	45.7	
Yes	11.0	4.0	34.0	
**Radiotherapy**				0.380
No	17.8	1.9	28.7	
Adjuvant	22.1	5.6	44.8	
Initial	11.8	5.6	38.6	
**Chemotherapy**				< 0.001
No	20.8	5.9	46.8	
Adjuvant	17.0	6.4	33.6	
Concurrent	11.0	5.6	30.5	
Initial	1.0	1.0	6.1	
**Year of admission**				< 0.001
1992–1995	12.8	5.2	100.8	
1996–2000	11.6	6.2	53.4	
2001–2005	16.9	5.4	36.4	

^a^p value was estimated using Log rank test.

### Risk factors for recurrence

We further analyzed the risk factors for cervical cancer recurrence by constructing Cox proportional hazards regression models (Table 3). We observed that miscarriage, clinical stage, and treatment received remained significant after adjustment for confounders. More specifically, the recurrence risk for subjects with three or more times of miscarriages was increased by 65% compared to those without a history of miscarriages (95% CI 1.23–2.22); the HR for subjects with clinical stage II was 1.73 (95% CI 1.24–2.41) and clinical stage III or IV was 2.04 (95% CI 1.32–3.16) compared to those with stage I; subjects received chemotherapy only were 3.62 times more likely to had recurrence compared to those received surgery only (95% CI 1.13–11.62). The sensitivity analysis that excluded subjects with recurrence within 3 months after discharge identified similar risk factors (Supplemental Table 3). When only including subjects with clinical stage I and II, we found that age greater than 60 years old was associated with a lower risk of recurrence (HR 0.53, 95% CI 0.30–0.94) than those with age younger than 40 years old; subjects with more than two times of miscarriages (HR 1.74, 95% CI 1.26–2.39) and received chemotherapy only (HR 5.61, 95% CI 1.58–19.95) had a higher risk of recurrence (Supplemental Table 4).

**Table 3:** Analyzing factors associated with cervical cancer recurrence using Cox regression models.

Variables	Unadjusted model			Adjusted model		
	HR	95% CI	*p* value	HR	95% CI	*p* value
**Age group (years)**						
< 40	Ref	–	–	Ref	–	–
40–59	0.95	0.76,1.20	0.679	0.99	0.76,1.29	0.935
≥ 60	0.67	0.45,0.99	0.045	0.61	0.37,1	0.052
**Parity (times)**						
0–2	Ref	-	-	Ref	-	-
≥ 3	0.75	0.61,0.92	0.007	0.87	0.67,1.12	0.284
**Miscarriage (times)**						
0	Ref	–	–	Ref	–	–
1–2	1.25	0.99,1.58	0.056	1.17	0.91,1.5	0.226
≥ 3	1.61	1.22,2.13	0.001	1.65	1.23,2.22	0.001
**Clinical stage**						
I	Ref	–	–	Ref	–	–
II	1.37	1.09,1.72	0.007	1.73	1.24,2.41	0.001
III/IV	1.59	1.15,2.19	0.005	2.04	1.32,3.16	0.001
**Pathological type**						
Squamous cell carcinoma	Ref	–	–	Ref	–	–
Adenocarcinoma	0.98	0.63,1.53	0.945	0.88	0.52,1.48	0.620
Others	1.68	0.87,3.26	0.125	1.94	0.71,5.29	0.193
**Level of differentiation**						
Well	Ref	–	–	Ref	–	–
Moderate	1.09	0.78,1.53	0.600	1.01	0.71,1.44	0.946
Poor/undifferentiated	1.49	0.86,2.58	0.152	1.41	0.78,2.52	0.253
**Treatment plan**						
Surgery only	Ref	–	–	Ref	–	–
Surgery plus adjuvant therapy	2.62	1.34,5.13	0.005	1.66	0.73,3.76	0.225
Radiotherapy only	2.26	1.15,4.46	0.019	1.35	0.56,3.25	0.503
Chemotherapy plus radiotherapy	4.69	2.34,9.38	< 0.001	1.94	0.8,4.7	0.144
Chemotherapy only	10.83	4.49,26.14	< 0.001	3.62	1.13,11.62	0.03
**Year of admission**						
1992–1995	Ref	–	–	Ref	–	–
1996–2000	1.82	1.13,2.92	0.013	1.9	1.14,3.18	0.014
2001–2005	3.02	1.94,4.68	< 0.001	3.05	1.86,4.98	< 0.001

*HR* hazard ratio, *CI* confidence intervals.

### Survival after recurrence

**Figure 3:**
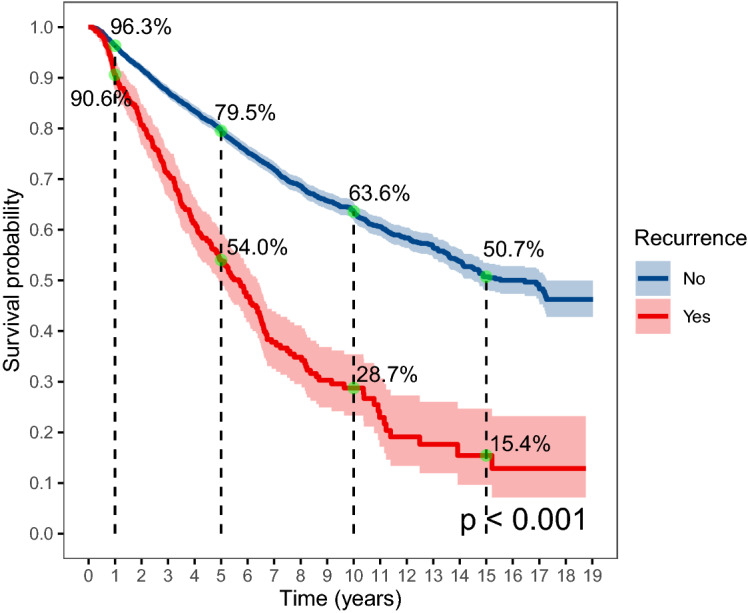
The overall survival probability by recurrence.

The total survival time was plotted for subjects with and without cancer recurrence (Fig. 3). The 1-year survival probability for those without cancer recurrence was 96.3%, the 5-year survival probability was 79.5%, and the survival probability dropped to 50.7% after 15 years of follow-up. The survival probability was significantly lower for those with cancer recurrence, which was 90.6% in the first year, 54.0% in the fifth year, 28.7% in the 10th year, and only 15.4% in the 15th year. The Cox regression showed that the HR of mortality was 2.79 (95% CI 2.42–3.22) for subjects with recurrence compared to those without recurrence after adjustment for confounders (Table 4). We also found that pathological type and year of admission were significantly associated with mortality in the adjusted model, and subjects with adenocarcinoma showed a higher risk for mortality compared to subjects with squamous cell carcinoma (HR 1.50, 95% CI 1.23–1.83). The sensitivity analysis including subjects with clinical stage I and II also found that recurrence, pathological type, and year of admission was associated with mortality (Supplemental Table 5).

**Table 4:** Analyzing factors associated with mortality using Cox regression models.

Variables	Unadjusted model			Adjusted model		
	HR	95% CI	*p* value	HR	95% CI	*p* value
**Recurrence**						
No	Ref	–	–	Ref	–	–
Yes	2.75	2.39,3.16	< 0.001	2.79	2.42,3.22	< 0.001
**Parity (times)**						
0–2	Ref	–	–	Ref	–	–
≥ 3	1.17	1.06,1.29	0.002	1.10	0.99,1.22	0.09
**Miscarriage (times)**						
0	Ref	–	–	Ref	–	–
1–2	0.97	0.87,1.08	0.546	0.97	0.87,1.09	0.625
≥ 3	0.98	0.85,1.12	0.732	1.01	0.87,1.17	0.936
**Pathological type**						
Squamous cell carcinoma	Ref	–	–	Ref	–	–
Adenocarcinoma	1.47	1.23,1.77	< 0.001	1.50	1.23,1.83	< 0.001
Others	1.22	0.84,1.78	0.293	1.66	0.98,2.84	0.061
**Level of differentiation**						
Well	Ref	–	–	Ref	–	–
Moderate	0.93	0.8,1.08	0.335	1.01	0.86,1.17	0.937
Poor/undifferentiated	1.1	0.83,1.45	0.524	1.15	0.86,1.52	0.347
**Year of admission**						
1992–1995	Ref	–	–	Ref	–	–
1996–2000	0.87	0.75,1	0.05	0.89	0.76,1.03	0.113
2001–2005	0.75	0.65,0.86	< 0.001	0.76	0.65,0.88	< 0.001

*HR* hazard ratio, *CI* confidence intervals.

## Discussion

In this study, we reported the longitudinal pattern of cumulative recurrence rate over 15 years for patients with cervical cancer after the first treatment. We found that the overall crude recurrence rate was 8.5%, and the median time to recurrence was 15.5 months. Also, we found that the cumulative recurrence rate increased rapidly from 3.8% in the first year to 8.0% in the fifth year, and the increasing trend slowed down afterward where the rate was 9.7% in the 10th year and 10.8% in the 15th year. The recurrence rate reported in this study was consistent with a systematic review in 2009 which found that the recurrence rate for patients who completed primary therapy for cervical cancer ranged from 8.0 to 26%, and the median time to recurrence ranged from 7 to 36 months^10^. A more recent cohort study from Denmark that enrolled a population of 1523 women with clinical stage I cervical cancer in 2005–2013 showed a recurrence rate of 6.4% after 5 years of follow-up^6^. A study in China reported that rates of recurrence were 16.9% and 12.4% for 148 stage IIb patients with radical hysterectomy and 290 stage IIb patients with radical radiotherapy, respectively^12^. The variation of recurrence rate reported in different studies may be explained by the composition of patients with different disease severity in each study and different treatments received. Note that the major strength of this study was the analysis of the long-term recurrence rate pattern for a large population over 15 years of follow-up, and we included subjects ranging from FIGO stage Ia to stage IVb, based on medical records of patients admitted to the hospital during 1992 to 2005. These findings may provide evidence for designing treatment and follow-up guidelines for patients with cervical cancer.

Our study reported several risk factors for cervical cancer recurrence, including younger age, lower parity, a greater number of miscarriages, higher clinical stage, lymph node metastasis, and received chemotherapy, and we found that miscarriage, clinical stage, and treatment received remained significant in the adjusted Cox regression model. These risk factors identified have also been reported by previous studies^4,20^. Clinical stage was the most commonly reported risk factor for cancer recurrence^19,21,22^, our study results showed that patients with clinical stage II had 1.73 times the risk of recurrence, and patients with stage III or IV had 2.04 times the risk compared to those with clinical stage I. Patients with advanced clinical stages had a greater range of cancer lesions, and a higher probability of peripheral invasion and lymph node metastasis, thus even if the patient was systematically treated, the possibility of recurrence for these patients was relatively higher^19,23^. In this study, we observed that patients with younger age had a higher risk of recurrence in the unadjusted model, while an insignificant association was observed in the adjusted model. It is still controversial whether age is related to recurrence with some studies reported positive associations^6,24^ and some reported negative associations^14,25^. It’s possible that patients with younger age had better adherence to the follow-up schedule, thus they were more likely to have a recorded recurrence. Unfortunately, we did not have detailed records on the adherence to follow-up, and it is important to further explore the reason between age and cancer recurrence. In addition, we did not include some risk factors that have been reported in previous studies, such as smoking, age of first intercourse, HPV infection, parametrial invasion, and vascular invasion^26–28^, and future investigations should take a more comprehensive considerations on related risk factors.

In this study, we observed that the treatment received varied by clinical stages, which jointly influenced the cancer recurrence rate. We found that surgery with adjuvant therapy was the most common treatment received for patients with clinical stage I, and more than half of the patients in clinical stage II and above were mainly treated with radiotherapy alone or chemotherapy plus radiotherapy. The treatment received in this study was generally consistent with the treatment guidelines indicating that surgery was the main treatment option for patients with early-stage cervical cancer^29^, and supplemental adjuvant therapy was added based on whether the patient has a risk factor for recurrence^30^. It’s also important to note that this study included a heterogeneous population with large variations in clinical stages and treatment plans, and we observed that the treatment plan was not consistent with the standard guidelines for a very small number of patients. For example, a few patients in Clinical stage I and II received chemotherapy only without surgery or radiotherapy. One possible explanation was that these patients were not able to receive surgery due to other complications or economic reasons. It is also possible that some patients were transferred to other local hospitals of whom the treatment regimen was not recorded in great detail. In addition, as this study collected data over a long period, we showed that the recurrence rate and survival rate increased during the follow-up period. The increase in recurrence rate may be due to the improved patient management system so that an increased number of subjects were followed up routinely and recurrence was more likely to be recorded. The improved survival rate may result from the improved standard of care over the years^31–34^. Note that in recent years, cisplatin-based chemotherapy and radiotherapy for advanced cervical cancer and inoperable early cervical cancer have been widely used in clinical practice, which has been shown to improve patient prognosis^35,36^.

In this study, we also demonstrated the overall survival rate for subjects with and without cancer recurrence separately and showed that cancer recurrence significantly reduced the overall survival rate, especially over the long term. The 5-year survival probability for patients with and without recurrence was 54.0% and 79.5%, and the 10-year survival probability was 28.7% and 63.6%, respectively. Such differences in survival between patients with and without recurrence were observed in many previous studies^6,37^, and these findings re-emphasized the importance of prevention of cancer recurrence in the management of cervical cancer.

Our study had several limitations. First, it was performed on patients in one hospital in South China, which may limit the ability to extrapolate these findings to the different settings. Also, the database is only available until 2010, and it would be necessary to conduct follow-up studies to update the recurrence rate data using more recent and comprehensive data records. Despite that, this study had a relatively large sample size with 5–19 years of follow-up, which provided a rare opportunity to study the overall trend of long-term recurrence rate for cervical cancer patients. Second, a small number of patients were lost to follow-up, which may lead to loss to follow-up bias, although the number has been kept to minimal as we have conducted several rounds of follow-up with multiple tracing methods. Third, despite the large overall sample size, we were not able to conduct multiple stratification analyses due to limited sample size after stratification, and we combined patients in clinical stages III and IV due to the small number of subjects in Stage IV. Fourth, we found that a very small proportion of subjects had a short disease-free time of fewer than 3 months after completion of the primary treatment, and these patients may have a disease progression rather than cancer relapse. However, we conducted a sensitivity analysis that excluded these subjects and reported consistent findings in the Cox regression.

In this study, we reported the pattern of cumulative recurrence rate using a study with 5–19 years of follow-up. The overall cumulative recurrence rate was 3.8% in the first year and increased to 8.0% in the fifth year, then the recurrence rate stayed at 9.7% in the 10th year and 10.8% in the 15th year. The median time to recurrence in this study was 15.5 months. Also, we reported that younger age, lower parity, a greater number of miscarriages, higher clinical stage, lymph node metastasis, and received chemotherapy were significantly associated with cancer recurrence. Patients with recurrence have a much poorer prognosis in terms of survival probability compared to those without recurrence. This study may provide important evidence for designing targeted interventions for cervical cancer treatment.

## Material and methods

### Study population

A retrospective cohort study was conducted for patients with cervical cancer that received cancer treatment from a cancer specialist hospital in Hunan Province, China from January 1992 to December 2005. The inclusion criteria were as follows: (1) subject was diagnosed with cervical cancer for the first time and had a clear clinicopathological diagnosis report; (2) subject had completed initial treatment in the study hospital and had a complete medical record; (3) subject was able to record the condition independently or with help to complete the follow-up activities; (4) age of the subject was greater than 18 years old. The exclusion criteria included: (1) subject had cognitive or communication dysfunction, or was unable to cooperate with the investigation; (2) subject was pregnant or had other severe diseases, including severe heart failure, renal failure, deep vein thrombosis, severe peripheral neuropathy, severe arterial insufficiency, other malignancies, etc.; (3) subject and family members did not cooperate with the recruitment process.

Informed written consent was obtained from all participants. Ethical approval was obtained from the Ethics Committee of Xiangya School of Public Health, Central South University, and all research was performed in accordance with the Declaration of Helsinki.

### Data collection and follow-up

Baseline information was collected by trained doctors through face-to-face interviews, phone calls, and the extraction of medical records. The trained doctor was responsible for filling in a questionnaire that included baseline characteristics (i.e. age, gravidity, parity, and history of miscarriage), clinicopathological characteristics (i.e. duration of discomfort before diagnosis, clinical stage, pathological type, and level of differentiation), and treatment plan (i.e. surgical method, radiotherapy method, and chemotherapy method). Clinical stage was classified according to the 2009 International Federation of Gynecology and Obstetrics (FIGO 2009) staging criteria, and lymph node metastasis was classified into Yes or No based on postoperative pathological biopsy results. The pathological type and degree of differentiation were obtained according to the pathological examination report. Treatment received referred to the treatment received after the first diagnosis, which was classified into the following categories: surgery only, surgery plus adjuvant therapy, radiotherapy only, concurrent chemotherapy and radiotherapy, and chemotherapy only. The treatment plan was decided by the physician-in-charge according to the standard treatment guidelines based on the patient’s condition and willingness for treatment. The post-treatment follow-up for subjects was planned for every three to 6 months during the first 5 years, and once a year afterward according to treatment guidelines in China^38^. Follow-up for subjects’ recurrence in this study was conducted every year through telephone, mail, and extraction of medical records by trained researchers after the patient was discharged from the hospital until December 31, 2010. Subjects lost to follow-up were censored, and subjects completely lost to follow-up were excluded from the data analysis. Cervical cancer recurrence was defined based on clinical–pathological diagnosis results, and this study was focused on the first recurrence of cervical cancer after the first treatment.

### Statistical analysis

Descriptive statistics were used for patient characteristics and summarized as mean $\mp$ SD or percentages. Differences in baseline characteristics and clinicopathological characteristics and treatment plan of the study participants with and without recurrent cervical cancer during follow-up were examined using student t-tests or chi-square tests, as appropriate. Average time to recurrence was presented with median and interquartile range (IQR) and a Log-rank test was conducted. Kaplan-Meier survival curves were used to plot the recurrence rate and survival rate after recurrence, stratified by different risk factors. Cox proportional hazards regression was used to calculate the hazard ratio (HR) and 95% confidence intervals (95% CIs) for cervical cancer recurrence and mortality. Proportional hazards assumptions were tested using Schoenfeld tests (Supplemental Table 2). Univariate analyses were conducted to evaluate patient characteristics associated with cervical cancer recurrence and mortality. The final multivariate model was selected based on the univariate analysis and biological significance, and variance inflation factors were used to test the collinearity between variables. Age, clinical stage, and treatment received were excluded from Cox regression for mortality due to violation of proportional hazards assumptions. A sensitivity analysis was conducted using Cox regression on cancer recurrence by excluding subjects with recurrence within 3 months as these subjects may have a disease progression rather than cancer relapse. We also conducted sensitivity analyses among subjects with clinical stage I and II using Cox regressions to explore factors associated with cancer recurrence and overall mortality. All statistical analyses were conducted using R version 4.0.5.

## Supplementary Information

Supplementary Information.

## Data Availability

The datasets used and/or analyzed during the current study are available from the corresponding author upon reasonable request.
