# Supplementary material for: Cervical cancer prognosis and related risk factors for patients with cervical cancer: a long-term retrospective cohort study
Source: Sci Rep. 2022 Aug 17;12:13994. doi: 10.1038/s41598-022-17733-8 (PMC9385852; doi:10.1038/s41598-022-17733-8)
Supplement: Supplementary file 1 — Supplementary Information. [file 41598_2022_17733_MOESM1_ESM.pdf]

Cervical cancer prognosis and related risk factors for patients with cervical cancer: a long-term retrospective cohort study

Authors' names: Jina Li[1†], Gaoming Liu[2†], Jiayou Luo[1], Shipeng Yan[2], Ping Ye[1], Jie Wang [1], Miyang Luo[1]

Authors' affiliations: [1] Xiangya School of Public Health, Central South University, Changsha, Hunan, China, 410008

[2] Hunan Cancer Hospital & The Affiliated Cancer Hospital of Xiangya School of Medicine, Central South University, Changsha, Hunan, China, 410013

Corresponding author:

Miyang Luo, PhD

Xiangya School of Public Health, Central South University

No. 238 Shang Ma Yuan Ling Road, Changsha, China, 410008

Phone: (+86) 0731-84805465

Email: miyangluo@csu.edu.cn

[†] These authors contributed equally to this work

Supplemental Table 1. Comparison of recurrence rate for different treatment received by clinical stages

	Clinical stage I		Clinical stage II		Clinical stage III/IV	
Treatment received	N (%)	Recurrence rate (%)	N (%)	Recurrence rate (%)	N (%)	Recurrence rate (%)
Surgery only	157 (8.1)	6 (3.8)	9 (0.5)	0 (0.0)	1 (0.2)	0 (0.0)
Surgery plus adjuvant therapy	1628 (84.2)	118 (7.2)	293 (17.3)	45 (15.4)	21 (4.0)	2 (9.5)
Radiotherapy only	110 (5.7)	13 (11.8)	1065 (62.9)	69 (6.5)	341 (65.1)	26 (7.6)
Chemotherapy plus radiotherapy	34 (1.8)	1 (2.9)	309 (18.3)	40 (12.9)	151 (28.8)	21 (13.9)
Chemotherapy only	5 (0.2)	0 (0.0)	16 (0.9)	4 (25.0)	10 (1.9)	2 (20.0)

[a] 204 missings.

Supplemental Table 2. Schoenfeld tests for proportional hazards assumptions

	Chi-square	df	*P* value
	Cox regression for recurrence		
Age group	0.136	2	0.934
Parity	2.8441	1	0.092
Miscarriage	0.0633	2	0.969
Clinical stage	2.1943	2	0.334
Pathological type	4.3831	2	0.112
Level of differentiation	1.8158	2	0.403
Treatment received	4.2679	4	0.371
Year of admission	1.1509	2	0.562
Global test	18.7195	17	0.345
	Cox regression for mortality		
Recurrence	1.173	1	0.279
Parity	0.115	1	0.735
Miscarriage	3.451	2	0.178
Pathological type	1.048	2	0.592
Level of differentiation	1.242	2	0.537
Year of admission	5.963	2	0.051
Global test	13.159	10	0.215

Supplemental Table 3. Sensitivity analysis for cancer recurrence excluding subjects relapsed within three months after discharge using Cox regression models (n = 4298)

Variables	Unadjusted model			Adjusted model		
	HR	95%CI	*p*-value	HR	95%CI	*p*-value
Age group (years)						
< 40	Ref	-	-	Ref	-	-
40–59	0.99	0.77,1.27	0.939	1.03	0.77,1.37	0.866
≥ 60	0.65	0.42,1.02	0.059	0.6	0.35,1.03	0.063
Parity (times)						
0–2	Ref	-	-	Ref	-	-
≥3	0.75	0.6,0.94	0.012	0.86	0.65,1.13	0.266
Miscarriage (times)						
0	Ref	-	-	Ref	-	-
1–2	1.29	1,1.66	0.052	1.21	0.93,1.59	0.158
≥ 3	1.68	1.24,2.27	0.001	1.71	1.24,2.35	0.001
Clinical stage						
I	Ref	-	-	Ref	-	-
II	1.45	1.13,1.86	0.003	1.71	1.18,2.46	0.004
III/IV	1.72	1.22,2.43	0.002	2.05	1.28,3.28	0.003
Pathological type						
Squamous cell carcinoma	Ref	-	-	Ref	-	-
Adenocarcinoma	1.08	0.68,1.71	0.760	1.00	0.58,1.72	0.999
Others	1.58	0.75,3.35	0.230	2.46	0.9,6.71	0.078
Level of differentiation						
Well	Ref	-	-	Ref	-	-
Moderate	1.06	0.74,1.52	0.752	1.01	0.7,1.47	0.944
Poor/undifferentiated	1.19	0.63,2.26	0.594	1.26	0.66,2.42	0.486
Treatment plan						
Surgery only	Ref	-	-	Ref	-	-
Surgery plus adjuvant therapy	2.84	1.33,6.07	0.007	2.07	0.76,5.62	0.152
Radiotherapy only	2.63	1.22,5.66	0.013	1.87	0.65,5.37	0.247
Chemotherapy plus radiotherapy	5.26	2.4,11.51	<0.001	2.66	0.92,7.71	0.071
Chemotherapy only	5.39	1.58,18.41	0.007	1.94	0.35,10.87	0.452
Year of admission						
1992–1995	Ref	-	-	Ref	-	-
1996–2000	1.94	1.17,3.2	0.010	2.09	1.22,3.58	0.007
2001–2005	2.88	1.79,4.62	<0.001	2.96	1.76,4.98	<0.001

HR, hazard ratio; CI, confidence intervals

Supplemental Table 4. Sensitivity analysis for cancer recurrence among subjects with clinical stage I and II using Cox regression models (n = 3630)

Variables	Unadjusted model			Adjusted model		
	HR	95%CI	*p*-value	HR	95%CI	*p*-value
Age group (years)						
< 40	Ref	-	-	Ref	-	-
40–59	0.89	0.69,1.14	0.358	0.96	0.72,1.27	0.759
≥ 60	0.59	0.37,0.94	0.027	0.53	0.3,0.94	0.031
Parity (times)						
0–2	Ref	-	-	Ref	-	-
≥3	0.78	0.62,0.98	0.033	0.98	0.74,1.29	0.862
Miscarriage (times)						
0	Ref	-	-	Ref	-	-
1–2	1.16	0.9,1.51	0.258	1.15	0.88,1.52	0.311
≥ 3	1.68	1.24,2.28	0.001	1.74	1.26,2.39	0.001
Pathological type						
Squamous cell carcinoma	Ref	-	-	Ref	-	-
Adenocarcinoma	1.03	0.63,1.69	0.891	0.94	0.54,1.63	0.818
Others	1.63	0.73,3.67	0.235	2.37	0.88,6.42	0.089
Level of differentiation						
Well	Ref	-	-	Ref	-	-
Moderate	1.03	0.71,1.49	0.888	1.01	0.68,1.49	0.966
Poor/undifferentiated	1.63	0.89,2.98	0.114	1.55	0.84,2.86	0.163
Treatment plan						
Surgery only	Ref	-	-	Ref	-	-
Surgery plus adjuvant therapy	2.43	1.07,5.48	0.033	1.71	0.76,3.88	0.197
Radiotherapy only	1.96	0.85,4.48	0.113	2.07	0.89,4.82	0.093
Chemotherapy plus radiotherapy	3.85	1.63,9.07	0.002	2.91	1.23,6.89	0.015
Chemotherapy only	7.33	2.07,25.99	0.002	5.61	1.58,19.95	0.008
Year of admission						
1992–1995	Ref	-	-	Ref	-	-
1996–2000	1.67	0.98,2.83	0.06	1.78	1.01,3.14	0.046
2001–2005	3.07	1.89,5.01	<0.001	3.00	1.75,5.14	<0.001

HR, hazard ratio; CI, confidence intervals

Supplemental Table 5. Sensitivity analysis for mortality among subjects with clinical stage I and II using Cox regression models (n = 3630)

Variables	Unadjusted model			Adjusted model		
	HR	95%CI	*p*-value	HR	95%CI	*p*-value
Recurrence						
No	Ref	-	-	Ref	-	-
Yes	2.89	2.46,3.38	<0.001	2.92	2.48,3.45	<0.001
Parity (times)						
0–2	Ref	-	-	Ref	-	-
≥3	1.13	1.01,1.27	0.032	1.09	0.96,1.23	0.176
Miscarriage (times)						
0	Ref	-	-	Ref	-	-
1–2	1.01	0.89,1.14	0.886	1.02	0.89,1.15	0.815
≥ 3	1.05	0.89,1.23	0.568	1.05	0.89,1.24	0.533
Pathological type						
Squamous cell carcinoma	Ref	-	-	Ref	-	-
Adenocarcinoma	1.51	1.23,1.86	<0.001	1.57	1.26,1.97	<0.001
Others	1.36	0.87,2.11	0.174	1.67	0.89,3.13	0.109
Level of differentiation						
Well	Ref	-	-	Ref	-	-
Moderate	0.93	0.78,1.1	0.376	1.01	0.85,1.21	0.916
Poor/undifferentiated	1.12	0.81,1.55	0.491	1.14	0.82,1.57	0.446
Year of admission						
1992–1995	Ref	-	-	Ref	-	-
1996–2000	0.8	0.68,0.94	0.006	0.77	0.65,0.92	0.003
2001–2005	0.76	0.65,0.89	0.001	0.73	0.62,0.86	<0.001

HR, hazard ratio; CI, confidence intervals
